# Bedtime negative affect, sleep quality and subjective health in rural China

**DOI:** 10.1186/s12889-024-17779-5

**Published:** 2024-01-23

**Authors:** Jiyao Sun, Nan Zhang, Jackie Carter, Bram Vanhoutte, Jian Wang, Tarani Chandola

**Affiliations:** 1 Social Statistics, Manchester Institute for Collaborative Research On Ageing (MICRA), The University of Manchester, HBS Building, Oxford Road, Manchester, M13 9PL UK; 2 Cathie Marsh Institute for Social Research (CMI), The University of Manchester, HBS Building, Oxford Road, Manchester, M13 9PL UK; 3 Center for Health Management and Policy Research, School of Public Health, Cheeloo College of Medicine, Shandong University, Jinan, 250012 China; 4 NHC Key Lab of Health Economics and Policy Research, Shandong University), Jinan, 250012 China; 5 École de Santé Publique, Université Libre de Bruxelles, Route de Lennik 808 - CP591, 1070 Brussels, Belgium; 6 Faculty of Social Sciences, University of Hong Kong, Pok Fu Lam, Hong Kong, China

**Keywords:** Subjective wellbeing, Negative affect, Health, Sleep quality, Fixed effect

## Abstract

**Background:**

The overall level of negative affect (NeA) has been linked to impaired health. However, whether the diurnal timing of NeA matters and whether the NeA-health relationship is mediated by sleep quality remain unclear.

**Methods:**

Using a longitudinal dataset (2006, 2009 and 2014 waves) consisting of 1959 participants, we examined the within-person impact of both bedtime NeA and non-bedtime NeA measured by Day Reconstruction Method (DRM) on subjective health measured by Visual Analogue Scale (VAS), and the mediating effect of sleep quality on the NeA-health relationships by fixed effect models.

**Results:**

Bedtime NeA predicted poorer health, while non-bedtime NeA was unrelated to health. The deleterious impact of bedtime NeA reduced and became non-significant after sleep quality was controlled for. Bedtime NeA also significantly predicted impaired sleep quality.

**Conclusions:**

Bedtime NeA is a stronger predictor of poorer health than non-bedtime NeA, and the deleterious influence of bedtime NeA on health seems to operate through poor sleep quality. Therefore, interventions to reduce bedtime NeA could potentially improve subsequent sleep quality, thereby protecting people to some extent from impaired health status.

## Introduction

Negative affect (NeA), one of the integral components of affective subjective wellbeing (SWB) [1], is associated with a wide range of adverse physical and mental health outcomes. For instance, stress and depression can increase the risk of premature mortality, coronary heart disease, disability, and other chronic disorders [2]. Fear and anxiety are related to phobias and other anxiety disorders and may also compromise immune functioning and create susceptibilities to stress-related physical disorders when combined with acute and chronic stress [3, 4]. Daily worry has been shown to be associated with heart rate volatility (e.g., high heart rate), which is a physiological risk factor for cardiovascular disease [5]. Anger and its poor management are associated with the etiology of heart disease [6] and some cancers [7]. Severe sadness and grief can also result in immunosuppression [4], and even suicide [8].

Prior research examining the NeA-health relationship has focused exclusively on an overall level of NeA. In these studies, overall NeA was commonly measured by asking a single retrospective question, like “During the past 12 months, would you say that you experienced a lot of stress, a moderate amount of stress, relatively little stress, or almost no stress at all?” [9], or using rating scales, such as the Positive and Negative Affect Schedule, where people rate the intensity of NeA over a specific period of time [10]. However, it should be noted that first, the lengthy reporting period used for assessing affective feelings is likely to produce data that are more analogous to global affective SWB than to more momentary affective states (which we used in this study), and the reported global affective states might be biased by several factors, such as the person’s current mood, personality traits, the sequence of questions, and limited recalled memories [11]. Second, the global retrospective measurements of NeA also shed no light on the relationship between time information over the day and the intensity of affective states, so the effect of the diurnal timing of NeA on health has been overlooked. This is a potentially serious neglect, largely because, as Kahneman et al. [12] mentioned, the quantitative information about time throughout the day and the intensity of affective states is potentially useful for assessing the burden of various diseases and the health consequences of negative emotions; investigating some social and environmental stressors; and evaluating policies and interventions. Therefore, research on the effect of the diurnal timing of NeA on health could also be meaningful and conducive to creating a finer-grained profile of the NeA-health relationship than only aggregating the NeA score into an overall level over a long period of time. However, to our best knowledge, no reported evidence directly looked at the effect of the diurnal timing of NeA on health.

Regarding the effect of the diurnal timing of NeA on health, NeA occurring at bedtime is likely to play a more critical role in shaping health than non-bedtime NeA, and sleep might mediate the NeA-health relationship. For example, research by Lancee et al. [13] among 64 participants from Netherlands compared the effects of daytime and nighttime sleep-related worry (e.g., I worried about the amount of sleep I am going to get) on sleep impairment, and found that nighttime sleep-related worry was significantly related to impaired sleep (e.g., difficulty falling asleep), while daytime sleep-related worry was not associated with sleep impairments. This indicates that nighttime NeA might play a more important role in predicting sleep-related problems and disrupting recovery process compared to daytime NeA. It is acknowledged that sleep plays a vital role in brain function and systemic physiology including appetite regulation, metabolism, and the functioning of immune, hormonal, and cardiovascular systems [14]. Impaired sleep and disrupted circadian rhythm by bedtime NeA could then cause various damaging consequences to health. For instance, poor sleep quality has been shown to be associated with impaired immune and metabolic function, obesity, mood disorders, cardiovascular disease and other chronic diseases, and increased risk of mortality [15, 16]. However, no published evidence has simultaneously examined in one study the effect of the diurnal timing of NeA on health by analyzing whether bedtime NeA is a more important determinant of poorer health than non-bedtime NeA, as well as the mediating role of sleep quality in the NeA-health relationship.

Much of the research that attempts to demonstrate the NeA-health relationship has been cross-sectional rather than longitudinal [9, 10]. The cross-sectional models might restrict the ability to identify the potential causal direction underlying the NeA-health association due to unobserved between-person heterogeneity (e.g., personality traits) [17]. ﻿Ambrona and López-Pérez [18] conducted a longitudinal analysis of the relationship between NeA and health wherein physical health was subsequently measured one month and one year later after NeA. However, Delongis et al. [17] argued that in such research designs, the time between the initial measurement of NeA and the subsequent measurement of health status was widely spaced and not monitored, it is therefore difficult and perhaps impossible to disentangle what has been going on not only psychobiologically, but also environmentally. Therefore, Delongis et al. [17] suggested that it might be better and useful to use a within-person (intraindividual) model of analysis rather than an across-person (interindividual) model with multiple measures of NeA levels and health obtained over time when examining the NeA-health relationship. In a longitudinal, within-person analysis, it is possible to observe whether fluctuations in NeA levels covary with changes in health ﻿with the effects of between-person differences being eliminated. Based upon above, the fixed effect model (which was used in this study) may be an appropriate approach to achieve the goal of examining the longitudinal, within-person NeA-health relationship. One of the superiorities of fixed effect models over other statistical approaches (e.g., random effect models) is that using each individual as his or her own control, it controls for easily measured variables such as gender, race, ethnicity, and region of birth, as well as for other variables that are difficult to measure such as intelligence, personality trait, and genetic makeup; thus, the bias in the estimate of the NeA-health relationship may be reduced [19].

For relevant SWB studies from China, most of them either exclusively used cross-sectional data and focused on reported evaluative SWB (e.g., life satisfaction and global happiness) [20, 21], overlooking the impact of affective SWB (e.g., negative emotions) on health, or only used health as one of the control variables in statistical models when examining the association between SWB and other aspects such as income [22]. To our knowledge, no prior studies have investigated the longitudinal, within-person NeA-health relationship, as well as the mediating effect of sleep quality, in China. The present study targeted rural Chinese for several reasons. First, according to the latest census, rural Chinese comprised over one-third (36.11%) of the entire Chinese population in 2021 [23], but the current SWB literature provides limited evidence regarding this subgroup. An analysis of Chinese SWB that excludes the rural residents is therefore far from comprehensive. In addition, it is acknowledged that China has a remarkable rural–urban disparity, in terms of the socioeconomic status and the entitlement to social welfare [24], which has been shown to have long term consequences in terms of mental wellbeing [25, 26]. Compared with their urban counterparts, Chinese rural dwellers are living in greater socioeconomic disadvantage. Conducting such research among rural Chinese can provide some evidence to shift policy priorities to improve rural living standards, health status and quality of life.

In brief, this study set out to examine the effects of the diurnal timing of NeA (both bedtime NeA and non-bedtime NeA) on subjective health, and the mediating role of sleep quality in the NeA-health relationship, using a longitudinal, within-person design in rural China. We hypothesize that in rural China, bedtime NeA is a stronger determinant of poorer health than non-bedtime NeA, and the deleterious effects of NeA on health would be mediated by sleep quality.

## Data and Methods

### Data

This study used panel data from the cooperative project “The evaluation of SWB based on the Day Reconstruction Method (DRM) in rural China” between Shandong University, China, and Harvard University, the United States [11]. This is a unique longitudinal dataset including three survey waves spanning eight years from 2006 to 2014, which investigated various aspects of rural life and the multidimensional constructs of SWB (both affective and evaluative SWB) of rural residents in China.

The panel dataset was collected in July and August of 2006, 2009 and 2014, and timed to avoid intensive farming seasons, such as harvest or planting. In July and August of 2006 and 2009, three provinces of China were selected: Shandong, An’hui, and Sichuan, according to levels of socioeconomic development and geographic location. Four counties were chosen from these provinces: Caoxian and Chiping (Shandong), Linquan (An’hui), and An’yue (Sichuan). These counties were also selected based on a combination of factors, including their socioeconomic development, geographic location, and representation of different demographic characteristics. Within each county, a stratified, multistage cluster random sampling design was used to select townships and villages. First, four townships were chosen based on levels of socioeconomic development and geographic location from each county; second, four villages were selected in a similar manner from each township. Subsequently, systematic sampling was employed to choose households based on the *Hukou* (household) registration in the villages; 25–30 households were visited within each village. In July and August of 2014, only Caoxian and Chiping were further surveyed in a same manner. Chinese rural dwellers aged 18 and older were interviewed face-to-face by trained interviewers in each household. There were 2847 respondents in 2006 at wave 1; 2748 respondents in 2009 at wave 2; 1385 respondents in 2014 at wave 3. Sample members who participated in at least two waves were included in the analysis of this study. The analytical sample, thus, comprises 1959 respondents, of which 470 participated in three waves; 926 in wave 1 and wave 2; 198 in wave 1 and wave 3; and 365 in wave 2 and wave 3. All participants were asked to provide written informed consent.

### Variables

#### Measure of subjective health

A Visual Analogue Scale (VAS) was used to measure respondents’ subjective health status on a vertical scale where the endpoint 0 represents “The worst health you can imagine” and 100 indicates “The best health you can imagine”. Respondents were asked to comprehensively assess their health status on the day of the survey and then marked a point on the scale [21]. VAS was included in the models as a continuous variable with higher values representing better health status.

#### Measure of bedtime NeA and non-bedtime NeA

The abbreviated DRM questionnaire from World Health Organization (WHO) was used to investigate participants’ both bedtime NeA and non-bedtime NeA. This approach has been validated in the Study on Global Ageing and Adult Health (SAGE) [27], and has been shown to have adequate reliability and construct validity [28], temporal stability (test–retest) [29], and measurement invariance [30] in previous studies. At each wave, the participants were randomly assigned to three different sets (A, B, and C) of the DRM questionnaire. In sets A, B, and C, participants reconstructed only a portion of their previous day’s activities from the morning when they woke up, from the afternoon when they had lunch, and from the evening when they had dinner, respectively, and responded to questions about each episode, including the type of activity (e.g., eating, shopping), the time spent on each activity, interacting partners (e.g., alone, with spouse), the friendliness felt towards the interacting partners (e.g., very friendly, a little irritated), and seven affective feelings they experienced about each activity (worried, rushed, irritated/angry, depressed, tense/stressed, calm/relaxed, and enjoying), which were reported on a 3-point scale (1 = not at all, 2 = a little, and 3 = very much).

##### Bedtime NeA

At each wave, participants kept reconstructing previous day’s episodes until they arrived at the activity “went to sleep for the night”, or when 15 min of interview time had elapsed in a continuous activity-by-activity manner. Bedtime NeA was calculated among those who reported their last activity “went to sleep for the night” based on the five negative feelings they experienced about the activity right before their last activity “went to sleep for the night”. Bedtime NeA was defined as the average of the scores given to the five negative feelings (worried, rushed, irritated/angry, depressed, and tense/stressed), and it ranged from 1 to 3 with higher values representing stronger bedtime NeA.

##### Non-bedtime NeA

Non-bedtime NeA was calculated among the same people who reported bedtime NeA and constructed in two steps at each survey wave [31]. First, during each non-bedtime activity, people’s *activity negative affect score*, which was the average of scores given to the five negative feelings (worried, rushed, irritated/angry, depressed, and tense/stressed), was calculated per specific activity to represent respondents’ negative feelings for that non-bedtime activity. Then, each *activity negative affect score* was weighted with the duration of that activity and aggregated over all recalled non-bedtime activities during the preceding day. The time-weighted aggregated *activity negative affect score* of an individual yielded the non-bedtime NeA score. In this study, non-bedtime NeA ranged from 0 to 2.8 with larger values representing higher non-bedtime negative feelings.

#### Measure of sleep quality

One item adapted from the Pittsburg Sleep Quality Index (PSQI) [32] was used to measure respondents’ subjective sleep quality, which reads, “How would you rate your last night’s sleep quality overall?” Responses were coded as 1 = Good, 2 = Moderate, and 3 = Poor. Sleep quality was treated as an ordinal categorical variable in models.

#### Measure of covariates

Three sets of the DRM questionnaire were included as covariates: 1 = morning set, 2 = afternoon set, and 3 = evening set.

Sociodemographic factors were included as covariates: Age (continuous); the highest education level completed (1 = no formal education, 2 = primary school, 3 = middle school and above); marital status (1 = married, 2 = other); occupation (1 = farmer and other (﻿this category represents participants who had multiple occupations not only as farmers, but also as workers, businessman, teachers, and village cadres, etc.), 2 = farmer, 3 = non-farmer (this category represents participants who were not farmers, such as workers, village cadres, businessman, teachers, students, and the unemployed, etc.)). The wealth of participants was evaluated with the International Wealth Index (IWI) [33], and was treated as a continuous variable in models with larger values indicating better economic status.

### Statistical analysis

#### Fixed effect regression model

As aforementioned, in this study, fixed effect regression models were conducted to examine the longitudinal, within-person relationships among bedtime NeA, non-bedtime NeA, sleep quality and subjective health over survey waves. In a fully-adjusted model, the fixed effect model of the present study for individual *i* = 1, …, N who was observed at several time periods *t* = 1, …, T, could be specified as follows:

$$\begin{array}{c} {Y}_{it}={\alpha }_{i}+{B}_{1}{NeA}_{it,bedtime}+{B}_{2}{NeA}_{it,non-bedtime}+{B}_{3}{sleep}_{it}+{B}_{4}{TC}_{it}+{u}_{it}\\ {\alpha }_{i}={B}_{0}+{B}_{k}{Z}_{i} \end{array}$$

where ${Y}_{it}$ is the subjective health status (VAS scores) at survey wave *t* (*t* = 1, 2, 3) of an individual *i*; ${B}_{0}$ is the intercept; ${Z}_{i}$ is the unobserved time-invariant heterogeneities (e.g., intelligence, personality trait, and genetic makeup, etc.) across the individuals *i*; ${B}_{k}$ is the coefficient for ${Z}_{i}$; ${\alpha }_{i}$ is the individual-specific intercepts that capture heterogeneities across individuals, and can be considered as the fixed effect of individual *i*; ${B}_{1}$ is the coefficient for bedtime NeA; ${B}_{2}$ is the coefficient for non-bedtime NeA; ${B}_{3}$ is the coefficient for sleep quality; ${B}_{4}$ is the coefficient for ﻿all time-variant covariates including sets of DRM questionnaire, age, highest education level completed, marital status, occupation, and IWI; ${u}_{it}$ is the error term [19].

#### Mediation analysis

**Fig. 1:**
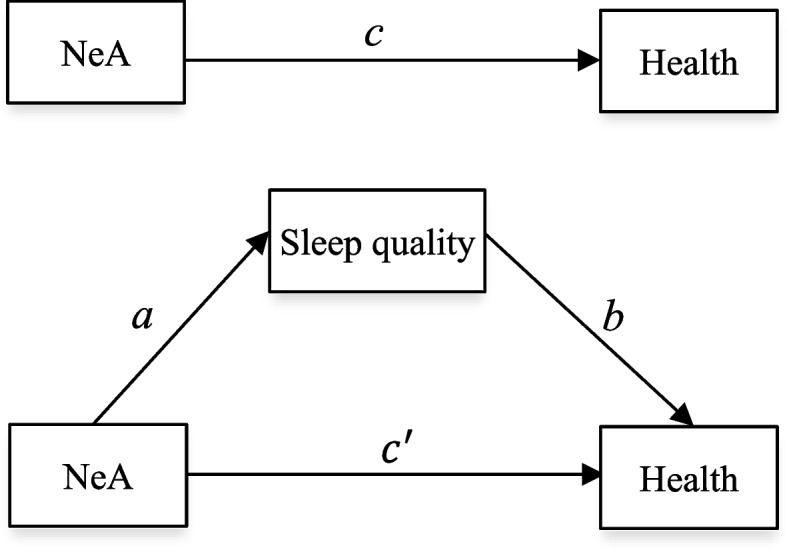
The path diagram for the mediating effect of sleep quality on the NeA-health relationship

To test for the mediating effect of sleep quality on the NeA-health relationship, we followed the commonly-used three-step mediation criteria developed by Baron and Kenny [34]. Specifically, mediation of sleep quality occurs if *Step 1* bedtime NeA and non-bedtime NeA significantly predict subjective health in the absence of sleep quality (path *c*); *Step 2* bedtime NeA and non-bedtime NeA significantly predict sleep quality (path *a*); and *Step 3* sleep quality significantly predicts subjective health when controlling for bedtime NeA and non-bedtime NeA (path *b*), and the effect of bedtime NeA and non-bedtime NeA on subjective health shrinks (from *c* to ${c}{\prime}$) upon addition of sleep quality (Fig. 1). It is worth noting that, since sleep quality was treated as an ordinal categorical variable, the fixed-effects ordered logit model was employed to test for *Step 2* (path *a*) [35].

## Results

### Descriptive statistics for respondents

Of all 1959 respondents, over half of the respondents were female (60.59%). The average baseline age was 48.11 (SD = 11.07). Table 1 showed that the lowest bedtime NeA and non-bedtime NeA were found in 2014 and 2009 with values of 1.06 (SD = 0.23) and 0.64 (SD = 0.40) respectively. The highest VAS score was found in 2009 (73.25, SD = 19.29). From 2006 to 2014, ﻿the vast majority reported good sleep quality with proportions ranging from 78.80% to 83.88%. Table 1 also showed the descriptive statistics for other sociodemographic covariates.

**Table 1:** Descriptive statistics for respondents from 2006 to 2014

Characteristics	2006	2009	2014
	Mean (SD)	Mean (SD)	Mean (SD)
Bedtime negative affect	1.08 (0.25)	1.08 (0.25)	1.06 (0.23)
Non-bedtime negative affect	0.65 (0.41)	0.64 (0.40)	0.74 (0.38)
VAS scores^a^	71.76 (20.92)	73.25 (19.29)	71.70 (20.46)
Age	48.25 (10.90)	51.27 (11.07)	55.76 (11.20)
IWI^b^	45.09 (12.72)	50.68 (12.86)	63.01 (12.08)
	N (%)	N (%)	N (%)
Sleep quality			
Good	1310 (82.75)	1467 (83.88)	814 (78.80)
Moderate	136 (8.59)	133 (7.6)	117 (11.33)
Poor	137 (8.65)	149 (8.52)	102 (9.87)
Highest education level completed			
No formal education	570 (35.80)	595 (33.86)	326 (31.56)
Primary school	468 (29.40)	531 (30.22)	333 (32.24)
Middle school and above	554 (34.80)	631 (35.91)	374 (36.21)
Marital status			
Married	1526 (95.73)	1673 (95.06)	974 (94.47)
Other	68 (4.27)	87 (4.94)	57 (5.53)
Occupation			
Farmer	1185 (74.34)	1319 (74.94)	683 (66.12)
Farmer and other	232 (14.55)	209 (11.88)	131 (12.68)
Non-farmer	177 (11.10)	232 (13.18)	219 (21.20)
Total sample size	1594	1761	1033

^a^*VAS* Visual Analogue Scale (0 = worst health, 100 = best health)

^b^*IWI* International Wealth Index

### Examining the associations among bedtime NeA, non-bedtime NeA, sleep quality and subjective health

Table 2 showed the results of *Step 1* and *Step 3* of the mediation analysis. We conducted four fixed effect models for VAS values. Model 1 examined the effect of bedtime NeA on the within-individual changes in subjective health across survey waves. Results showed that when controlling for time-variant covariates, bedtime NeA was significantly and negatively associated with health (path *c*). Specifically, for a given individual, as bedtime NeA increased across waves by one unit, VAS decreased by 6.53 units (*p* < 0.05).

**Table 2:** Results of fixed effect models for the associations among bedtime NeA, non-bedtime NeA, sleep quality and subjective health in rural China from 2006 to 2014

Variables	VAS values							
	Model 1		Model 2		Model 3		Model 4	
	Coefficient	SE^a^	Coefficient	SE	Coefficient	SE	Coefficient	SE
Bedtime negative affect	-6.53*	2.62			-5.86*	2.65	-4.18	2.70
Non-bedtime negative affect			-3.94	2.08	-3.16	2.11	-2.64	2.11
Sets of DRM (Ref. Morning set)^b^								
Afternoon set	1.98	2.66	1.71	2.68	1.62	2.67	1.72	2.65
Evening set	2.93	2.62	0.56	2.91	1.04	2.91	1.29	2.89
Age	-0.56 *	0.24	-0.49*	0.24	-0.53*	0.24	-0.49*	0.24
Highest education level completed (Ref. No formal education)								
Primary school	5.38 *	2.49	5.32 *	2.49	5.26 *	2.48	5.53 *	2.47
Middle school and above	8.52 *	3.48	8.55 *	3.49	8.32 *	3.48	8.46 *	3.46
Marital status (Ref. Married)								
Other	-4.50	4.37	-5.38	4.38	-4.82	4.37	-4.37	4.35
Occupation (Ref. Farmer and other)								
Farmer	-6.66 **	2.27	-6.88 **	2.28	-7.03 **	2.28	-7.42 **	2.27
Non-farmer	-3.29	2.60	-3.69	2.61	-3.59	2.61	-4.02	2.60
IWI^c^	0.03	0.08	0.03	0.08	0.04	0.08	0.05	0.08
Sleep quality (Ref. Good)								
Moderate							-4.49	2.51
Poor							-7.93 **	2.88
Number of observations	2183		2183		2183		2183	

^a^*SE* Standard error

^b^*Ref*. Represents the reference group.

^c^*IWI* International Wealth Index.

T Test: *** *p* $$<$$ 0.001, ** *p* $$<$$ 0.01, * *p* $$<$$ 0.05.

Model 2 examined the within-person impact of non-bedtime NeA on health across waves. Results showed that when controlling for time-variant covariates, non-bedtime NeA was unrelated to health (path *c*). Model 3 controlled for both bedtime NeA and non-bedtime NeA, and demonstrated consistent results that non-bedtime NeA was unrelated to health, whereas bedtime NeA was predictive of poor health over time (*p* < 0.05).

Model 4 further controlled for the mediator, sleep quality, on the basis of model 3, and showed that both coefficients for bedtime NeA and non-bedtime NeA were reduced; particularly, the impact of bedtime NeA on health became non-significant (path ${c}{\prime}$). Sleep quality was significantly associated with health in model 4 (path *b*); specifically, for a given individual, compared with good sleep quality, poor sleep quality contributed to a significant decrease in VAS with a value of 7.93 across waves (*p* < 0.01).

Additionally, to test for *Step 2* (path *a*), fixed-effects ordered logit models were conducted to examine the associations between bedtime NeA, non-bedtime NeA and sleep quality. Table 3 showed that bedtime NeA significantly contributed to poor sleep quality with the coefficient of 1.51 (*p* < 0.05), whereas there was no significant impact of non-bedtime NeA on sleep quality.

**Table 3:** Results of fixed-effects ordered logit models for the associations between bedtime NeA, non-bedtime NeA and sleep quality in rural China from 2006 to 2014

Variables	Sleep quality (1 = Good, 2 = Moderate, 3 = Poor)	
	Coefficient	SE^a^
Bedtime negative affect	1.51*	0.75
Non-bedtime negative affect	0.56	0.42
Sets of DRM (Ref. Morning set)^b^		
Afternoon set	0.60	0.66
Evening set	0.54	0.64
Age	0.08	0.05
Highest education level completed (Ref. No formal education)		
Primary school	0.49	0.44
Middle school and above	-0.27	0.69
Marital status (Ref. Married)		
Other	0.31	0.79
Occupation (Ref. Farmer and other)		
Farmer	-0.53	0.53
Non-farmer	-0.58	0.71
IWI^c^	0.01	0.02
Number of observations	296	

^a^*SE* Standard error.

^b^*Ref*. Represents the reference group.

^c^*IWI* International Wealth Index.

Z Test: *** *p* $$<$$ 0.001, ** *p* $$<$$ 0.01, * *p* $$<$$ 0.05.

In brief, the above findings suggest that bedtime NeA is a stronger predictor of poorer health than non-bedtime NeA, and the deleterious influence of bedtime NeA on health seems to be operating through poor sleep quality.

## Discussion

This study is amongst the first to examine the impact of the diurnal timing of NeA including bedtime NeA and non-bedtime NeA on subjective health, and the mediating role of sleep quality in the NeA-health relationship, using a longitudinal, within-person design in rural China. The core research questions addressed by this study were as follows: in rural China, (a) is bedtime NeA a stronger determinant of poorer health than non-bedtime NeA? (b) To what extent the effect of NeA on health can be mediated through sleep quality?

For the first research question, when only controlling for time-variant covariates (from model 1 to model 3), within-person analysis revealed that non-bedtime NeA was unrelated to health, while bedtime NeA was found to be predictive of health, suggesting that bedtime NeA is a stronger predictor of poorer health than non-bedtime NeA. No reported studies have directly analyzed the impact of the diurnal timing of NeA on health; however, some studies examining the relationship between the timing of cortisol (a biochemical marker of chronic stress) and health could provide some enlightenment. For example, a prospective study by Kumari et al. [36] in a sample from Whitehall II study demonstrated that bedtime cortisol was predictive of subsequent cardiovascular-related mortality, while waking cortisol levels were unrelated to subsequent mortality. Tene et al. [37] reported consistent results among 182 cognitively intact ischemic stroke patients that higher bedtime cortisol levels were associated with larger neurological deficits, brain atrophy, and worse cognitive results, while post-awakening cortisol levels were not associated with any neuroimaging findings or cognitive scores. The present study consistently mirrored the cortisol-health patterns found in previous studies [36, 37].

The second research question was to what extent the effect of NeA on health could be mediated through sleep quality. After further controlling for sleep quality, the coefficient for bedtime NeA was no longer statistically significant, suggesting that the deleterious effect of bedtime NeA on health was mediated by impaired sleep quality. However, the potential mechanism underlying this phenomenon warrants further empirical investigation. Studies have shown that NeA, such as stress, is associated with increased heart rate reactivity, increased systolic blood pressure, and reduced vagal tone [38, 39]. Reduced vagal tone has been shown to be associated with increased sleep disruptions and poor sleep quality [40, 41]. Bedtime NeA which occurred right before sleep could result in these cardiac autonomic changes but might leave people with no time to recover from these physiological changes before going to sleep. Therefore, compared with non-bedtime NeA, bedtime NeA was more likely to directly undermine sleep quality (as shown in Table 3) and recovery processes, such as by ﻿leading to heightened pre-sleep arousal. Sleep plays a critical role in brain function and systemic physiology, including metabolism, appetite regulation, and the functioning of immune, hormonal, and cardiovascular systems [14]. Disruption of circadian rhythm and sleep deprivation have been shown to accelerate the risk of ill health, and even mortality [42]. In such a case, the deleterious impact of bedtime NeA on health might be explained by impaired sleep, such as poor sleep quality in this study. However, this study sheds no light on the potential physio-biological mechanisms linking bedtime NeA, poor sleep quality, and undermined health; thus, the above explanations should be treated with caution.

This study suggests that the standard method of calculating the NeA score through a (diurnal) time-weighted aggregated activity negative affect score may not be as important for future health as the bedtime negative affect score. The standard time-weighted aggregated measure may under-estimate the effect of bedtime NeA on future health, which suggests researchers, at the very least, should give consideration to bedtime values of NeA, rather than solely relying on overall time-weighted measures of NeA. Therefore, another significance of this study is about the importance of measuring bedtime NeA separately from the standard time-weighted approach.

Our findings could inform future interventions to focus more on reducing bedtime NeA so as to protect and promote sleep quality, thereby improving health levels and quality of life. For example, research by Sun et al. [11] demonstrated that some social environmental factors, such as the type of activity (e.g., shopping, watching TV) and quality of social interaction, represented by the friendliness felt towards the interacting partners, have a significant effect on the diurnal rhythms of people’s affective experiences in daily life. It is possible that the affective feelings could change in response to stimulation from the environment. Therefore, improving some social environmental factors, such as organizing and participating in pleasant activities and creating a friendly interactive environment in daily life, could effectively contribute to the decline in NeA, especially when NeA is high at bedtime, thereby preventing people from impaired sleep quality and then improving health levels.

Several limitations should be considered. First, the data were collected from four counties of three provinces in China; thus, the findings might not be generalized to all rural Chinese or other populations. Second, the present study only took into account a limited number of variables to examine the NeA-health relationship. Some other factors, such as healthy behaviors, utilization of health service, and some physiological markers, might also need to be considered in order to gain a comprehensive understanding about the NeA-health relationship. Third, the present study only focused on the global self-rated health. Different dimensions of health status, such as ﻿the health-related somatic symptoms, might also need to be evaluated in order to depict a panorama of the NeA-health relationship. Fourth, the present study employed DRM to measure people’s NeA. However, as the DRM still to some extent relies on retrospective self-reports, it could possibly produce some general methodological problems. For example, individuals with higher levels of cognitive ability may be better able to reconstruct their day, whereas participants of lower ability may forget what they were doing or how they felt about the activity [43]. Fifth, there is a potential overlap between the measures of NeA and subjective health, while they tap into different dimensions: self-reported health—a measure of general health and well-being, and NeA—a measure of negative affect. For example, an individual experiencing a deterioration in mental health might report lower scores on both measures. This overlapping nature might introduce a challenge in discerning the distinct contribution of NeA to the observed health outcomes. Furthermore, while the longitudinal, within-person analysis employed in this study is suitable for discerning the potential causal direction underlying the NeA-health association, it is not without limitations. For example, although this approach can effectively handle time-invariant covariates (and confounders), it falls short in handling unobserved time-variant factors. Finally, the calculation of bedtime NeA was based on participants’ negative feelings they experienced about the activity right before their last activity “went to sleep for the night”. Given that participants may be involved in different bedtime activities with varying durations, there is no established standard to determine the timeframe considered as bedtime NeA.

## Conclusion

The present study uniquely sheds light on the effect of bedtime NeA and non-bedtime NeA on subjective health, and the mediating role of sleep quality in the NeA-health relationship using a longitudinal, within-person design in rural China. We found that bedtime NeA is a stronger predictor of poorer health than non-bedtime NeA, and the deleterious influence of bedtime NeA on health seems to operate through poor sleep quality. Therefore, interventions to reduce bedtime NeA could potentially improve subsequent sleep quality, thereby protecting people to some extent from impaired health status.

## Data Availability

All data analyzed during this research can be acquired from J.S. and J.W.

## Abbreviations

NeA: Negative affect
DRM: Day Reconstruction Method
VAS: Visual Analogue Scale
SE: Standard error
IWI: International Wealth Index
